# Cinnamaldehyde Restores Ceftriaxone Susceptibility against Multidrug-Resistant *Salmonella*

**DOI:** 10.3390/ijms24119288

**Published:** 2023-05-26

**Authors:** Lizi Yin, Yuhong Gou, Yuyun Dai, Tao Wang, Kexin Gu, Ting Tang, Sajjad Hussain, Xiaoli Huang, Changliang He, Xiaoxia Liang, Gang Shu, Funeng Xu, Ping Ouyang

**Affiliations:** 1 College of Veterinary Medicine, Sichuan Agriculture University, Huimin Lu 211, Chengdu 611130, China; 2 College of Animal Science and Technology, Sichuan Agriculture University, Huimin Lu 211, Chengdu 611130, China

**Keywords:** cinnamaldehyde, multidrug-resistant bacteria, cell membrane, extended-spectrum beta-lactamase

## Abstract

In recent years, infections caused by multidrug-resistant (MDR) bacteria have greatly threatened human health and imposed a burden on global public health. To overcome this crisis, there is an urgent need to seek effective alternatives to single antibiotic therapy to circumvent drug resistance and prevent MDR bacteria. According to previous reports, cinnamaldehyde exerts antibacterial activity against drug-resistant *Salmonella* spp. This study was conducted to investigate whether cinnamaldehyde has a synergistic effect on antibiotics when used in combination, we found that cinnamaldehyde enhanced the antibacterial activity of ceftriaxone sodium against MDR *Salmonella* in vitro by significantly reduced the expression of extended-spectrum beta-lactamase, inhibiting the development of drug resistance under ceftriaxone selective pressure in vitro, damaging the cell membrane, and affecting its basic metabolism. In addition, it restored the activity of ceftriaxone sodium against MDR *Salmonella* in vivo and inhibited peritonitis caused by ceftriaxone resistant strain of *Salmonella* in mice. Collectively, these results revealed that cinnamaldehyde can be used as a novel ceftriaxone adjuvant to prevent and treat infections caused by MDR *Salmonella*, mitigating the possibility of producing further mutant strains.

## 1. Introduction

*Salmonella* spp. are common pathogens that cause foodborne diseases in animals. In recent years, the development of drug resistance in *Salmonella* has become a challenge in food safety and clinical treatment. The Centres for Disease Control and Prevention (CDC) data revealed that an increasing proportion of non-typhoidal *Salmonella* strains are resistant to ceftriaxone [1]. Ceftriaxone, a third-generation cephalosporin antibiotic with good hydrolytic stability against a variety of β-lactamases, is generally used to treat severe salmonellosis. Ceftriaxone sodium can disrupt bacterial cell wall synthesis by targeting the cross-linking of penicillin-binding proteins and peptidoglycans [2]. The resistance of *Salmonella* to cephalosporin antibiotics is usually mediated by β-lactamases, which are divided into extended-spectrum β-lactamases (ESBLs), carbapenemases, and Amp C-type β-lactamases. Among these enzymes, ESBL production is involved in the induction of MDR bacteria [3]. Thus, the development of an alternative drug line to treat and control ESBL-producing pathogenic bacteria is urgently required.

The accumulation of antibiotic residues in body tissues poses a serious threat to human and animal health [4], and can lead to the development of antibiotic resistance and environmental pollution. Therefore, the use of antibiotics as feed additives is prohibited in many countries [5]. Pursuing systematic drug combination strategies, including antibacterial strategies, in the clinical settings has resulted in therapeutic success for HIV, cancers, and cardiovascular diseases, and many others [6]. Furthermore, the use of plant-derived bioactive compounds in combination with conventional antibiotics has been proposed as an effective method for controlling MDR bacteria.

Cinnamaldehyde, also known as 3-phenyl-2-propenal, is the main active ingredient of cinnamon oil (content > 75%) [7], and is used in traditional Chinese medicine. Because it is non-toxic and has low toxicity in the human body, it can be widely used as a food additive, preservative, or antibacterial food packaging material [8,9,10]. Researchs have shown that cinnamaldehyde has demonstrated synergistic interactions with various antibiotics against gram-positive and gram-negative bacteria [11,12,13,14,15]. The antibacterial activity of cinnamaldehyde, along with its combinatorial effects with traditional antibiotics, has not yet been tested against ceftriaxone resistant *Salmonella*. In the present study, we aimed to test the synergistic antibacterial activity of cinnamaldehyde and ceftriaxone sodium against various MDR bacteria with special focus on *Salmonella*.

## 2. Results

### 2.1. Cinnamaldehyde Enhances the Effects of Ceftriaxone Sodium against MDR Bacteria

**Figure 1:**
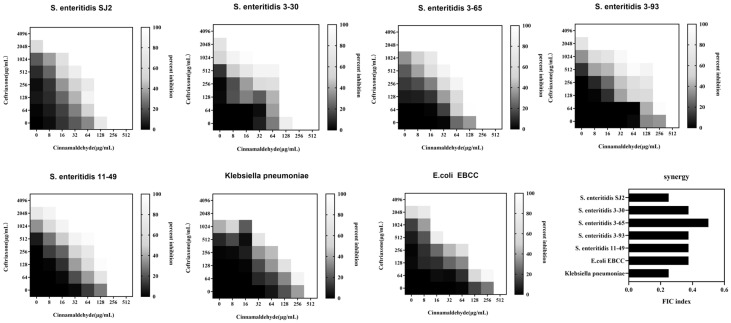
Cinnamaldehyde drastically potentiates ceftriaxone activity against various MDR Gram-negative bacteria. Checkerboard broth microdilution assays between cinnamaldehyde and ceftriaxone against *Salmonella* SJ2, 3–30, 3–65, 3–93, 11–49, *E. coli*, and *Klebsiella pneumoniae*. The lighter the blue, the stronger the synergistic inhibitory effect. Row X and Column Y represent the concentration of ceftriaxone and cinnamaldehyde. The FIC indices were calculated at one quarter of MICs of cinnamaldehyde. Synergy is defined as an FIC index of ≤0.5.

MDR *Salmonella* SJ2, which was isolated from duck and resistant to β-lactams (with TEM, SHV, CTX-M, OXA, and CMY β-lactamase-encoding genes), aminoglycosides, and tetracycline, was used in this study (Table 1). The MIC of cinnamaldehyde against the *Salmonella* SJ2 strain was 256 μg/mL, whereas that of ceftriaxone sodium was 4096 μg/mL. The combined inhibitory index of cinnamaldehyde and ceftriaxone sodium against SJ2 was FICI (fractional inhibitory concentration index) = 0.25 < 0.5, indicating their synergistic inhibitory activity (Figure 1).

To determine whether this combination was also applicable to other β-lactam-resistant Gram-negative bacteria, we evaluated the synergistic activity of the combined application of cinnamaldehyde and ceftriaxone on three other MDR Gram-negative bacteria, including drug-resistant *E. coli* and *Klebsiella pneumoniae*, using the checkerboard broth microdilution method. These three pathogens were resistant to ceftriaxone sodium, with MICs ranging from 1024–2048 μg/mL. We found that the combination of cinnamaldehyde and ceftriaxone sodium exerted synergistic inhibitory (FICI ≤ 0.5) effects (Figure 1), however these concentrations were still above the breakpoint (4 μg/mL) for these strains. Cinnamaldehyde is a potential adjuvant of antibiotics to reverse resistant bac-teria. Better active compounds will obtained by medicinal chemistry and pharmaceu-tical preparations.

**Figure 2:**
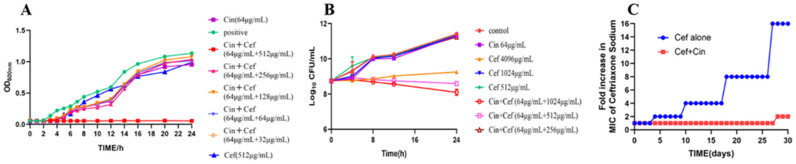
Cinnamaldehyde enhances the efficacy of ceftriaxone sodium and minimizes the induction of resistance. (**A**) The sub-inhibitory mass concentration of cinnamaldehyde (64 μg/mL) can cooperate with the sub-inhibitory mass concentration of ceftriaxone (512 μg/mL) to inhibit the proliferation of drug-resistant *Salmonella* within 24 h. (**B**) Time-dependent killing of pathogens by the combination of ceftriaxone sodium and cinnamaldehyde. *Salmonella* SJ2 cells were grown to exponential phases in MHB broth, then treated with PBS, ceftriaxone sodium (Cef, 512, 1024 or 4096 µg/mL) or cinnamaldehyde (Cin, 64 µg/mL) alone or in combination (Cin + Cef, 64 µg/mL + 256 µg/mL, 512 µg/mL or 1024 µg/mL). The bacterial CFUs/mL at different time points during 24 h were determined. All experiments were performed thrice, and the mean ± SD is shown. (**C**) The addition of cinnamaldehyde (32 μg/mL, one quarter of MIC) prevents the evolution of ceftriaxone resistance to *Salmonella* ATCC 14028 in vitro. Resistance acquisition during serial passaging in the presence of 0.25 × MIC levels of ceftriaxone (0.25 µg/mL).

To verify the synergistic inhibitory effect of cinnamaldehyde and ceftriaxone sodium on the proliferation of *Salmonella* SJ2, the growth curve of *Salmonella* SJ2 within 24 h of the combined treatment was generated. When the bacteria were grown in the presence of sub-inhibitory concentrations of cinnamaldehyde (64 μg/mL) alone, or in combination with ceftriaxone sodium (32–256 μg/mL), the growth of the strain was inhibited to a certain extent, but the effect was not significant, and it could not inhibit the proliferation of *Salmonella* SJ2. However, when cinnamaldehyde at a sub-inhibitory concentration was combined with 512 μg/mL ceftriaxone sodium, *Salmonella* SJ2 barely grew in 24 h. Hence, the proliferation of *Salmonella* SJ2 was inhibited when cinnamaldehyde at 1/4 MIC was combined with ceftriaxone sodium at 1/8 MIC (Figure 2A).

Next, a time-dependent cytotoxicity curve was used to observe whether different concentrations of ceftriaxone sodium or in combination with cinnamaldehyde inhibited bacterial proliferation when *Salmonella* grew to the logarithmic phase. As shown in Figure 2B, the sub-inhibitory concentration of cinnamaldehyde and bacteriostatic concentration of ceftriaxone sodium showed weak bactericidal activity against *Salmonella*. In contrast, when the sub-inhibitory concentration of cinnamaldehyde was combined with ceftriaxone sodium at a concentration of 512 μg/mL or higher, both showed significant bactericidal activity against *Salmonella*.

To better understand the effect of cinnamaldehyde on the resistance induced by ceftriaxone sodium, standard *Salmonella* strain ATCC 14028 was cultured in presence of sub-inhibitory concentration of ceftriaxone sodium (1/4 MIC) for 24 h in the presence or absence of cinnamaldehyde (32 μg/mL). Interestingly, we failed to obtain any resistant mutants in the combination group (Figure 2C). In contrast, the ceftriaxone sodium alone group produced highly resistant strains with a 16-fold increase in the MIC. Sub-inhibitory concentrations of antibiotics affect pathogenic microorganisms by regulating antibiotic tolerance and resistance [16]. These results suggest that cinnamaldehyde weakened the induction of *Salmonella* resistance by ceftriaxone sodium.

### 2.2. Cinnamaldehyde Affects the Cell Wall and Cytomembrane of MDR Salmonella

Our results demonstrated that cinnamaldehyde enhanced the inhibitory effect of ceftriaxone sodium against drug-resistant *Salmonella*. The antibacterial effects of ceftriaxone sodium are mainly attributed to the destruction of the bacterial cell wall and inhibition of cell wall synthesis. Studies have shown that the antibacterial effects of cinnamaldehyde against *Salmonella* are executed by affecting the bacterial cell membrane and cell wall [17]. Thus, we attempted to elucidate the potential mechanism underlying the synergistic effects of these two antibacterial agents.

**Figure 3:**
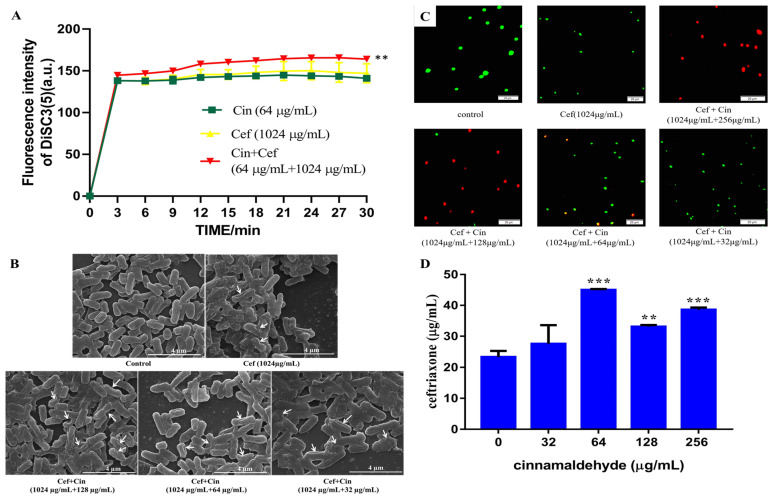
The sub-inhibitory concentration of cinnamaldehyde and the sub-inhibitory ceftriaxone act synergistically on the bacterial cell membrane. (**A**) The combination of sub-inhibitory concentration of cinnamaldehyde (64 μg/mL) and sub-inhibitory concentration of ceftriaxone (1024 μg/mL) depolarized the bacterial cell membrane. (**B**) The combination of sub-inhibitory concentration of cinnamaldehyde and sub-inhibitory concentration of ceftriaxone damaged the morphology of bacteria (white arrow showed the sunken of bacteria). (**C**) The combination of sub-inhibitory concentration of cinnamaldehyde and sub-inhibitory concentration of ceftriaxone made more bacteria die after damaging the bacterial cell membrane. (**D**) Cinnamaldehyde (32~256 μg/mL) significantly promotes the accumulation of sub-inhibitory concentration of ceftriaxone (1024 μg/mL) in bacteria. Note: ** means 0.001 < *p* < 0.01, *** means *p* < 0.001.

First, we tested the effects of cinnamaldehyde and ceftriaxone sodium on bacterial membrane permeability by performing a membrane depolarisation test. DiSC3(5) is a membrane potential-sensitive dye and emits a fluorescence when entering the aqueous solution containing the depolarised or damaged bacterial [18,19,20]. The depolarisation of the bacterial membrane upon treatment with the combination drugs could be detected by the change in fluorescence value. Meanwhile, fluorescence strength is an indicator of membrane damage. As shown in Figure 3A, the effect of cinnamaldehyde (64 μg/mL) or ceftriaxone sodium (1024 μg/mL) alone on bacterial membrane potential was not as significant as that exerted by the combination of the two drugs, indicating that the combination of cinnamaldehyde and ceftriaxone caused significant depolarisation and membrane damage.

To better understand the effect of the combination of cinnamaldehyde and ceftriaxone sodium on the bacterial membrane, scanning electron microscopy was performed to observe the changes in bacterial membranes after 12 h of drug administration (Figure 3B). In the blank control group, *Salmonella* was short and rod-shaped, the cell membrane had no obvious wrinkles, the surface was smooth, intercellular junctions were intact, and the cell morphology was distinguishable. After treatment with 1/4 MIC of ceftriaxone sodium, small number of bacteria had shrunken cell membranes, and cellular adhesions were observed. Treatment with 32 μg/mL cinnamaldehyde and 1/4 MIC of ceftriaxone showed that cell membranes were wrinkled in a small number of bacteria, but the degree of bacterial adhesion was increased. Furthermore, treatment with 64 μg/mL cinnamaldehyde and 1/4 MIC of ceftriaxone showed that the cell membranes of most bacteria collapsed, the degree of the collapse was severe, the adhesion between the cells was tight, and only a small part of the bacteria maintained their normal cellular architecture (short rod shape). Meanwhile, treatment with 128 μg/mL cinnamaldehyde and 1/4 MIC of ceftriaxone showed that the bacterial cell membranes were severely shrunk, the cells were tightly connected to a mass, the structure of *Salmonella* was destroyed, the cells were broken, and the cells with blurred boundaries could not maintain their normal shape. Notably, our previous studies showed that sub-inhibitory concentrations of cinnamaldehyde did not significantly damage the cell membrane of *Salmonella*. This shows that cinnamaldehyde caused more serious damage to the membrane and morphology of *Salmonella* owing to the synergistic effect in combination with ceftriaxone sodium, and the effect was concentration dependent.

Next, we used the live/dead cell staining method to observe changes in the number of bacteria after adding different concentrations of cinnamaldehyde and ceftriaxone sodium. As shown in Figure 3C, when the sub-inhibitory concentration of ceftriaxone sodium (1024 μg/mL) was used alone, there were almost no dead bacteria (stained red). However, with the addition of cinnamaldehyde, the number of dead bacteria gradually increased in a concentration-dependent manner, which proves that the combination of the two drugs increased the permeability of the bacterial membrane.

Sufficient intracellular accumulation of antibiotics is a prerequisite for antibacterial activity, particularly against Gram-negative pathogens [21,22]. We used the method described by Song et al. [23] and HPLC to quantitatively analyse the antibiotics accumulated in *Salmonella* SJ2 after pre-treatment with different concentrations of cinnamaldehyde and ceftriaxone sodium. Compared to the treatment with only the sub-inhibitory concentration of ceftriaxone sodium, we observed a significant concentration-dependent increased in the intracellular accumulation of antibiotics in bacteria (Figure 3D). This increased accumulation may be attributed to the impaired integrity of the bacterial membrane.

In summary, our findings indicated that cinnamaldehyde can restore the sensitivity of *Salmonella* to ceftriaxone sodium through various mechanisms, including membrane destruction and increased intracellular antibiotic accumulation.

### 2.3. The Effect of Cinnamaldehyde Combined with Ceftriaxone Sodium on Bacterial β-lactamase and Its Gene Expression

**Figure 4:**
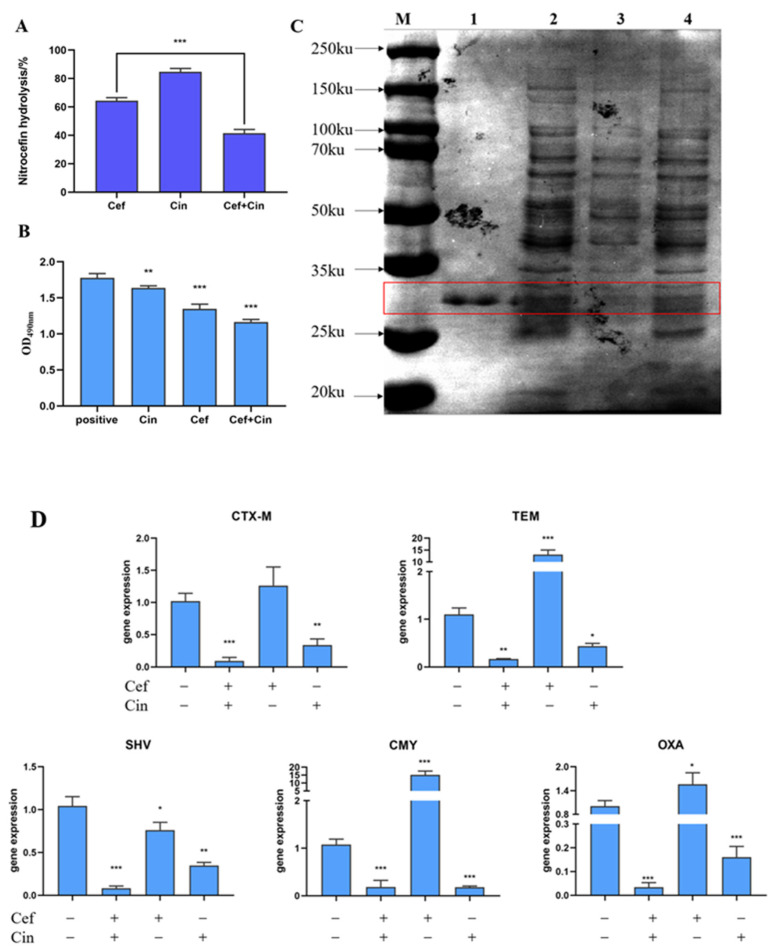
The effect of combination of cinnamaldehyde and ceftriaxone on β-lactamase. (**A**) The effect of sub-inhibitory concentration of cinnamaldehyde (64 μg/mL) and sub-inhibitory concentration of ceftriaxone (1024 μg/mL) on the hydrolysis rate of bacterial ESBL when used alone or in combination. (**B**) The effect of sub-inhibitory concentration of cinnamaldehyde and sub-inhibitory concentration of ceftriaxone on bacterial ESBL activity when used alone or in combination. (**C**) The combination of sub-inhibitory concentration of cinnamaldehyde and sub-inhibitory concentration of ceftriaxone inhibits the expression of bacterial ESBL. M: marker, 1: β-lactamase, 2: positive, 3: Cef + Cin (1024 μg/mL + 64 μg/mL), 4: Cin (64 μg/mL). (**D**) The combination of sub-inhibitory concentration of cinnamaldehyde (64 μg/mL) and sub-inhibitory concentration of ceftriaxone (1024 μg/mL) inhibits the expression of bacterial extended-spectrum β-lactamase resistance genes. Note: * means 0.01 < *p* < 0.05, ** means 0.001 < *p* < 0.01, *** means *p* < 0.001.

In this study, cephalosporin hydrolysis was used to detect the ESBL activity. Since the β-lactam ring of cephalosporins can be hydrolysed by β-lactamase, it is often used to detect β-lactamase. After culturing the bacterial cultures in the presence of cinnamaldehyde and ceftriaxone sodium, bacterial β-lactamase was extracted and used to detect the hydrolysis rate of bacterial β-lactamase after the combined treatment. The results are shown in Figure 4A. Compared with the control group, the hydrolysis rate of cephalosporin after combined treatment was significantly lower than that of the ceftriaxone sodium group alone.

SDS-PAGE was performed to detect changes in β-lactamase expression more intuitively, and pure β-lactamase was used as a positive control (Figure 4B). The molecular weight of β-lactamase is between 25–35 kDa. Compared to the bacterial group, the level of proteins in this molecular weight range was slightly reduced in the cinnamaldehyde group, although the decrease was not significant. Cinnamaldehyde exerted a weak inhibitory effect on β-lactamase expression. In contrast, ESBL expression in the combination group was significantly reduced or even disappeared. Thus, our results provide evidence that cinnamaldehyde can be combined with ceftriaxone sodium to reduce β-lactamase expression in drug-resistant *Salmonella.* Cinnamaldehyde (64 μg/mL) exerted an inhibitory effect on β-lactamase expression in in drug-resistant *Salmonella*. In our previous experiment [24], the proteomic results showed that cinnamaldehyde (64 μg/mL) significantly reduced β-lactamase content in *Salmonella* (*p* = 0.003). In contrast, soluble proteins content in the combination group was significantly reduced or even disappeared. Thus, our results provide evidence that cinnamaldehyde combined with ceftriaxone sodium can to reduce multiple soluble proteins content in drug-resistant *Salmonella*, and also possible reduced the β-lactamase expression.

Next, we studied whether treating the bacteria with a combination of cinnamaldehyde and ceftriaxone sodium inhibits the activity of bacterial β-lactamase. Treatment of cephalosporins with β-lactamase causes a colour change from yellow to red owing to β-lactamase hydrolysis. The higher the OD490 value is, the greater is the activity. Therefore, in this experiment, we examined the effect of combination medication on enzyme activity by comparing the changes in the OD490 value of each treatment. The results showed that the enzyme-containing extracts obtained from the control and cinnamaldehyde-alone groups were similar and higher than those obtained from the other two groups. The enzyme activity of the cinnamaldehyde-alone group was significantly lower than that of the control group. This showed that cinnamaldehyde exerted inhibitory effects on β-lactamase activity. However, the enzyme activity of the supernatant derived from the combined drug and ceftriaxone sodium groups was comparable and significantly lower than those of the other two groups. When ceftriaxone sodium is used alone, the enzyme hydrolyzes the β-lactam ring of ceftriaxone sodium, thereby decreasing the number of enzymes that react with ceftriaxone, resulting in a low OD value. The enzyme activity of the combination group was lower than that of the ceftriaxone sodium group alone, indicating that the synergistic effect of cinnamaldehyde and ceftriaxone sodium may have inhibited the enzyme activity (Figure 4C).

ESBL production is the main mechanism underlying bacterial resistance to β-lactam antibiotics. ESBLs are based on different genotypes and can be divided into five types: TEM, SHV, CTX-M, OXA, and others [25]. In this study, the expression of TEM, SHV, CTX-M, OXA, and CMY β-lactamase-encoding genes was evaluated after treatment with cinnamaldehyde or ceftriaxone sodium at sub-inhibitory concentrations, alone or in combination. We observed that compared with the antibiotic group alone, the combination treatment significantly reduced the expression of all β-lactamase-encoding genes that impart antibiotic resistance. Interestingly, when used alone, ceftriaxone sodium enhanced the expression of some β-lactamase-encoding genes (Figure 4D). Cinnamaldehyde significantly inhibited the expression of CMY, OXA, CTX-M and TEM (Figure 4D), and then reduced the ESBLs prodcution. One possible explanation is that when ceftriaxone sodium is used alone, it induces the overexpression of β-lactamase-encoding genes, and when used in combination with cinnamaldehyde, this induction is reversed, although elucidating the exact mechanisms warrants further investigation.

### 2.4. The Effects of Transcriptome Analyses of Cinnamaldehyde Acting on MDR Salmonella

**Figure 5:**
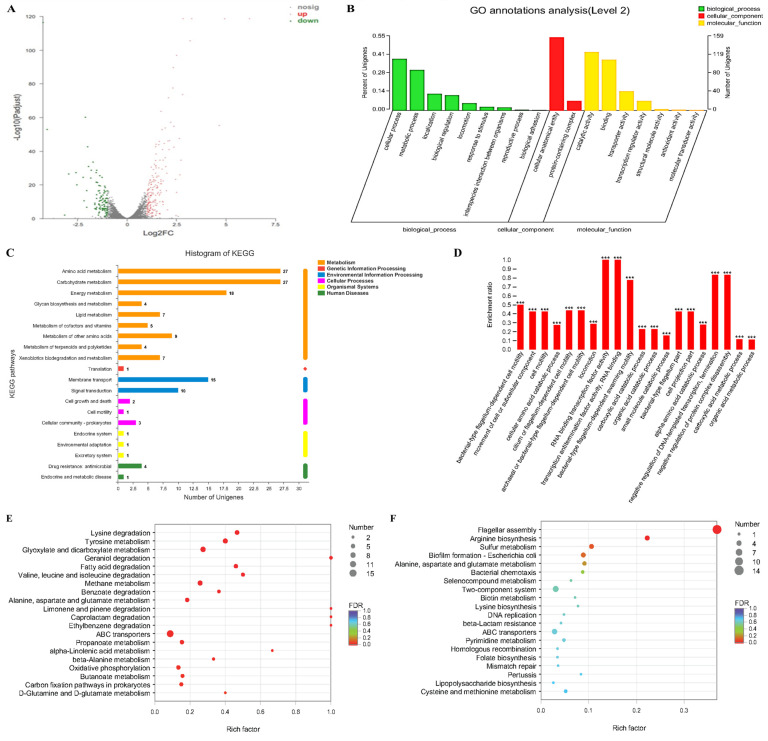
Transcriptome analysis of *Salmonella* SJ2 after exposure to ceftriaxone alone or the combination of ceftriaxone plus cinnamaldehyde. (**A**) Volcano plot and (**B**) GO (gene ontology) annotation analysis of the differential expression genes (DEGs) in *Salmonella* SJ2 after exposing ceftriaxone (1024 µg/mL) or the combination of ceftriaxone (1024 µg/mL) plus cinnamaldehyde (64 mg/mL). (**C**) KEGG (Kyoto Encyclopedia of Genes and Genomes) annotation pathway analysis. (**D**) GO enrichment analysis. (**E**) Differential genes up-regulated in KEGG enrichment analysis. (**F**) Differential genes down−regulated in KEGG enrichment analysis. Note: *** means *p* < 0.001.

To gain a deeper understanding of the molecular mechanisms underlying cinnamaldehyde-induced changes in gene expression at the mRNA level, we performed transcriptomic analysis of *Salmonella* SJ2 after treatment with a combination of ceftriaxone sodium and ceftriaxone. As shown in Figure 5A, compared with the group treated with ceftriaxone only, the group treated with cinnamaldehyde combined with ceftriaxone had 195 genes differentially upregulated and 114 genes differentially downregulated. Gene ontology (GO) annotation analysis showed that these differentially expressed genes (DEGs) were related to biological processes (such as cell and metabolic processes), cell components, and molecular functions (such as catalytic activity and binding) (Figure 5B). Catalytic activity and receptor activity can directly affect the effect and yield of bacterial toxins as well as bacterial pathogenicity [26].

The Kyoto Encyclopedia of Genes and Genomes (KEGG) is the main public database for analysing gene functions, linking genomic information with functional information, and is often used to explore RNA regulatory and metabolic pathways [27]. KEGG annotation pathway analysis is shown in Figure 5C. The pathways were classified into seven categories: metabolism, genetic information processing, environmental information processing, cellular processes, organismal systems, human diseases, and drug development. The results showed that most of the DEGs were concentrated in two types of metabolic pathways and environmental information processing, which were specifically related to amino acid metabolism, carbohydrate metabolism, energy metabolism, membrane transport, cell movement, and signal transduction pathways (Figure 5C).

The results of the GO enrichment analysis are shown in Figure 5D. DEGs were significantly enriched in multiple GO terms related to RNA-binding transcription, bacterial flagella-dependent movement, negative regulation of protein compound decomposition, and negative regulation of DNA transcription (Figure 5D).

The upregulated DEGs were significantly enriched in carbon cycle pathways and amino acid metabolism (Figure 5E), mainly in lysine degradation, tyrosine metabolism, glyoxylic acid and dicarboxylic acid metabolism, geraniol degradation, and fatty acid degradation. KEGG enrichment analysis showed that the downregulated DEGs were significantly enriched in flagellar assembly, arginine biosynthesis, and sulfur metabolism processes (Figure 5F).

### 2.5. Cinnamaldehyde Can Reverse the Resistance to Ceftriaxone In Vivo

**Figure 6:**
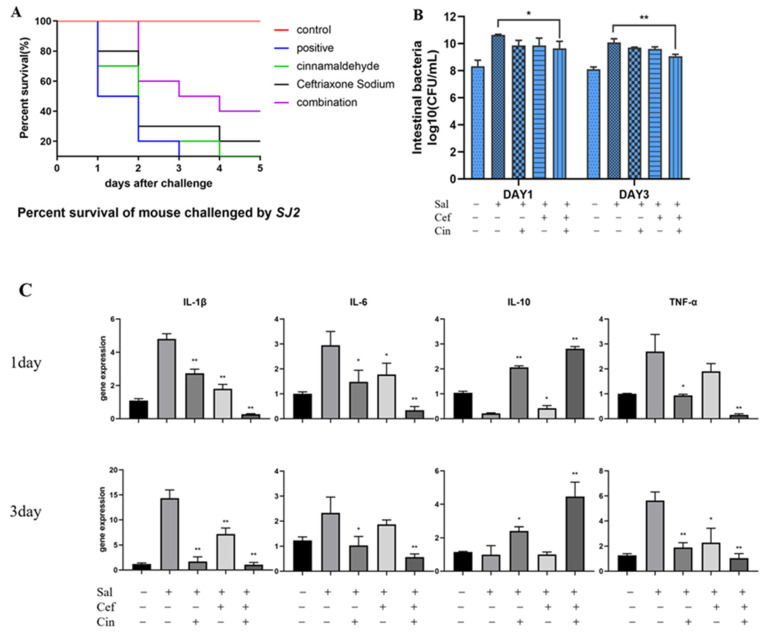
Cinnamaldehyde rescues ceftriaxone activity in vivo. (**A**) Survival rate of mice. (5 days) (**B**) During the acute infection period, observed number of bacteria in the cecum of different groups of mice. (**C**) During the acute infection period, the changes in the expression of cecal inflammatory factor genes in different groups of mice. Note: * means 0.01 < *p* < 0.05, ** means 0.001 < *p* < 0.01.

First, we established a mouse model of drug-resistant *Salmonella* SJ2 infection. As shown in Figure 6A, when the groups of mice were individually treated with ceftriaxone sodium and cinnamaldehyde, the survival rates of the mice were 20% and 0% within 5 days of infection, respectively. However, the survival rate of the combined drug group was 40%. In addition, the number of caecal bacteria in mice treated with the combination of cinnamaldehyde and ceftriaxone sodium was significantly lower than that in the single medication group (Figure 6B), indicating that cinnamaldehyde can help ceftriaxone sodium prevent *Salmonella* colonisation in the intestinal tract of infected mice. In addition, we detected the levels of intestinal inflammatory factors and evaluated gene expression changes in each group of mice, which were consistent with the results of the caecal bacterial load test. The combined medication group showed reduced levels of the inflammatory factors IL-6, IL-1β, and TNF-α in the intestinal tract of infected mice (Figure 6C).

The expression of the examined genes was down regulated in the combination group, while the expression of the IL-10 encoding gene was upregulated along with IL-10 content. This indicates that cinnamaldehyde can reverse resistance to ceftriaxone sodium in mice. Compared with the challenged group, cinnamaldehyde effectively improved the efficacy of ceftriaxone sodium in infected mice during the acute infection period (three days). The combination of cinnamaldehyde and ceftriaxone sodium is expected to treat infectious diseases caused by drug-resistant *Salmonella* spp.

## 3. Discussion

The emergence and rapid spread of antibiotic resistance in pathogenic bacteria poses a significant threat to public health worldwide [28]. Identifying new adjuvants that can restore the efficacy of antibiotics and improve the clinical treatment of infectious diseases is an effective strategy against resistant bacteria. Inhibitors of β-lactamases such as clavulanic acid have been widely used in clinical practice for decades [29]. Metformin can be used as a potential adjuvant for tetracycline to restore its antibacterial activity against MDR *E. coli* [30]. The China Agricultural University discovered that linear antimicrobial peptides can be used in conjunction with various antibiotics to synergistically inhibit MDR *E. coli* [23]. The mechanism of action of these two antibiotic adjuvants is related to the destruction of the integrity of the cell membrane, enhancement of the permeability of the cell membrane, and promotion of antibiotic accumulation.

Ceftriaxone sodium, a third-generation cephalosporin antibiotic, is one of the most widely used antibiotics for clinical antibacterial therapy [23,31,32]. However, studies have shown that the excessive use of ceftriaxone sodium can lead to resistance against *Salmonella* [33]. Hence, combination therapy may be a novel strategy. Ceftriaxone sodium is often used in combination with other antibiotics for the clinical treatment of enterobacterial infections and other diseases [34].

As an effective component against *Salmonella* infection, cinnamaldehyde does not act through a single mechanism. Instead, drugs affect the normal metabolic activities of bacteria and other microorganisms through multiple mechanisms. The antibacterial effect of cinnamaldehyde is related to its concentration, as it shows a dose-effect relationship [35]. A high concentration of cinnamaldehyde affects the distribution and interactions of fatty acids on the bacterial cell membrane [36], inhibits enzyme activity on the cell membrane, regulates the fluidity of the cell membrane, enhances its osmotic effect, and causes bacterial death. At moderate concentrations, cinnamaldehyde can inhibit the activity of ATP protease in the cell and affect cellular function and biofilm synthesis, thereby exerting a bacteriostatic effect [37]. At low concentrations, cinnamaldehyde can combine with intracellular proteins, hormones, and other factors to affect normal cell division [38,39]. Cinnamaldehyde has been studied in combination with other antibacterial monomeric compounds, such as eugenol. When the two are used in combination, they have a significant inhibitory effect on the formation and elimination of Listeria monocytogenes and *Salmonella* typhimurium [17]. Studies have also found that cinnamaldehyde can be combined with ciprofloxacin or cefotaxime to synergistically inhibit the expression of resistance genes in MDR K. pneumoniae, A. baumannii, and *E. coli* [15].

Since cinnamaldehyde has been proven to effectively inhibit *Salmonella* by affecting the cell membrane [40], and ceftriaxone sodium can destroy the bacterial cell wall, we believe that the synergistic antibacterial effects of these two drugs in combination may destroy the cellular structure to a greater extent. As evident from our study outcomes, treatment with a combination of cinnamaldehyde and ceftriaxone sodium depolarised the bacterial cell membrane and affected its integrity. We infer that, via this mechanism, cinnamaldehyde promotes the accumulation of ceftriaxone sodium in bacteria, which leads to increased and rapid death of bacteria. We also found that the combination of cinnamaldehyde and ceftriaxone sodium can significantly reduce ESBL content in bacteria and the overexpression of drug resistance-imparting genes, indicating that cinnamaldehyde can effectively reverse bacterial resistance to ceftriaxone sodium. This confirmed that cinnamaldehyde treatment can increase bacterial sensitivity to ceftriaxone sodium. Through transcriptome analysis, we also found that cinnamaldehyde may kill bacteria by forcing them to enter a highly metabolic state after promoting the accumulation of ceftriaxone sodium in the bacteria. Compared to the ceftriaxone sodium treatment group, cinnamaldehyde effectively reversed drug resistance in drug-resistant *Salmonella*, prevented bacterial colonisation in the intestinal tract of infected mice, inhibited the expression of inflammatory factors, and enhanced the efficacy of ceftriaxone sodium in infected mice during the acute infection period.

Flagella are filamentous accessory proteins located on the bacterial surface that mediate the movement and chemotaxis of bacteria in the external environment. It is also the main virulence factor of *Salmonella* and is associated with bacterial pathogenicity [41,42]. Downregulation of the flagellar assembly pathway can indirectly reduce *Salmonella* pathogenicity. Meanwhile, sulfur is an essential element for all organisms. The downregulation of sulfur metabolism downregulates the synthesis of sulfur-containing amino acids, which dysregulates the catabolism of sulfur-containing molecules in organisms. For instance, glutathione and its secondary metabolites build a protective barrier when microorganisms face external stress and exert antioxidant and detoxification effects [27]. However, when glutathione synthesis is downregulated, the organisms lose their diverse defense platforms. Lopatkin et al. (2021) found that many bacteria are forced to accelerate their metabolism when antibiotics are used for treatment, leading to the accumulation of toxic products, which damage bacterial cells and kill them. Thus, changes in core metabolic pathways may be a general mechanism of antibiotic resistance [43]. 

The development of multidrug resistance significantly compromises the efficacy of antibacterial therapies, particularly for Gram-negative bacterial and pathogen-associated infections [44,45]. Gram-negative bacteria have inherited and evolved multiple strategies, such as the unique impermeable outer membrane, to overcome antibiotic treatments [46,47]. The main antibacterial effect of cinnamaldehyde is attributed to the destruction of cell membrane integrity and permeability, and its synergistic mechanism with ceftriaxone sodium also depends on this attribute. On this basis, cinnamaldehyde can increase the intracellular concentration of ceftriaxone sodium and significantly reduce the expression of extended-spectrum β-lactamase genes by destroying the integrity of bacterial cell membranes.

In summary, cinnamaldehyde, a non-toxic food additive, exhibited a satisfactory biosafety profile. In view of its inhibitory effect on *Salmonella* drug resistance, it is a promising ceftriaxone sodium adjuvant that can help overcome the challenges associated with clinical MDR *Salmonella* infection. Our results should encourage researchers to study other compounds from known medicinal plant essential essential oils with synergistic effects as antibiotic adjuvants to potentially help alleviate the global public health problem of drug resistance.

## 4. Materials and Methods

### 4.1. Bacteria and Reagents

Bacterial strains were used in this study showed in Table 1. All clinical strains used in this study were preserved at the Pharmacy Laboratory in the School of Veterinary Medicine of Sichuan Agricultural University. The drug resistant Klebsiella pneumoniae was donated by the Affiliated Hospital of Zunyi Medical University. Unless otherwise noted, the strains were grown in Mueller-Hinton (MH) broth (MHB, Qingdao Hi-Tech Park Haibo Biological Technology Co., Ltd., Qingdao, China) or on MH agar (MHA) plates at 37 °C. Cinnamicaldehyde (>98% purity, HPLC; CAS No. 104-55-2) was purchased from Shanghai Macleans Biochemical Technology Co., Ltd. (Shanghai, China). A stock solution of cinnamic aldehyde (40.96 mg/mL) was prepared in dimethyl sulfoxide (DMSO). Ceftriaxone sodium was purchased from Sigma–Aldrich (St. Louis, MO, USA).

### 4.2. Minimum Inhibitory Concentration Assay

According to the CLSI 2015 guidelines, the microbroth dilution method was used to determine the minimum inhibitory concentration (MIC) of cinnamaldehyde and ceftriaxone sodium against drug-resistant Gram-negative bacteria. The MIC is defined as the lowest concentrations of drugs with no visible bacterial growth and resistance was defined when the MIC of ceftriaxone was greater than 4 μg/mL. Briefly, MH culture medium and bacterial suspension were added to the positive control group, and the culture medium, bacterial suspension, and drugs were added to the negative control group, whereas the blank control group contained only the culture medium. Subsequently, cultures from the positive, negative, and control groups were plated in a 96-well plate and cultured at 37 °C for 18 h. The positive control group was turbid, while the blank control and negative control groups were clear and transparent.

### 4.3. Checkerboard Assays

The synergistic activity of cinnamaldehyde and ceftriaxone sodium and the fractional inhibitory concentration (FIC) were measured using checkerboard assays. The dilution used in the drug combination was determined based on the MIC of the two antibacterial drugs, and six dilutions were selected. FIC index was calculated using the following formula [48]

$$\mathrm{FIC}\;\mathrm{index}=\frac{\mathrm{MICab}}{\mathrm{MICa}}+\frac{\mathrm{MICba}}{\mathrm{MICb}}=\mathrm{FICa}+\mathrm{FICb},$$

where a: cinnamaldehyde and b: ceftriaxone sodium. MICab with A = Concentration of Cinnamaldehyde in combination with Ceftriaxone sodium in a well. MICba with B = Concentration of Ceftriaxone sodium in combination with Cinnamaldehyde in the same well. An FIC index ≤ 0.5 indicates synergy [49].

### 4.4. Bacterial Growth Curves

As described previously [50], 50 μL of ceftriaxone sodium diluted with MH medium (128, 256, 512, 1024, 2048 μg/mL) was added to each well of a 96-well plate, and then 50 μL of cinnamaldehyde diluted with MH medium (final concentration 256 μg/mL), 90 μL MH medium, and 10 μL overnight bacterial culture (106 CFU/mL) were added. The plate was incubated at 37 °C for 0, 1, 2, 3, 4, 5, 6, 7, 8, 10, 12, 14, 16, 20, and 24 h. The absorbance in each well was read at 600 nm using a microplate reader (Thermo Scientific, Waltham, MA, USA). The absorbance of the blank control group was set to zero to generate the bacterial growth curve.

### 4.5. Time-Dependent Cytotoxicity Assay

The time-kill curve test was carried out according to the method of Md. Akil Hossain [51]. Overnight cultures of *Salmonella* SJ2 were diluted (1:10,000) in fresh MHB media and incubated for 8 h at 37 °C with shaking at 200 rpm. The culture was then incubated in samples containing PBS, ceftriaxone sodium (256, 512, 1024, or 4096 µg/mL), or cinnamaldehyde (64 µg/mL) alone or in combination. At 0, 4, 8, 12, and 24 h, 100 µL aliquots were collected, centrifuged, resuspended in PBS, and serially diluted. The dilutions were spotted on MHA plates, and colony counts were determined after overnight incubation at 37 °C. All experiments were performed in triplicates.

### 4.6. Analysis of Drug Resistance Development

*Salmonella* typhimurium (ATCC 14028) growing in the exponential phase was diluted (1:1000) in fresh MHB medium. Ceftriaxone sodium was added to the culture to obtain 1/4 of the MIC; cinnamaldehyde and ceftriaxone sodium were added to another flask to obtain 1/4 MIC for both. The flasks were incubated at 37 °C for 24 h, followed by the induction of the second generation according to the above method and continuous induction for 30 generations. At the same time, the MICs before induction and after each induction of the next five generations were measured and compared, and the fold increase in the two groups of ceftriaxone sodium treatment was analysed relative to the initial MIC [30]. The experiments were performed with biological replicates.

### 4.7. Membrane Depolarization Assay

Bacterial cells were washed and resuspended in 5 × 10^−3^ M HEPES (pH 7.0, plus 5 × 10^−3^ M glucose) to obtain an OD600 of 0.5. At 10 min, 3, 3-dipropylthiadicarbocyanine iodide (DiSC3 (5); Aladdin, Shanghai, China) (final concentration: 0.5 × 10^−6^ M) was added. After 30 min, cinnamaldehyde (final concentration, 64 μg/mL) or ceftriaxone sodium (final concentration, 1024 µg/mL) was injected. The membrane potential of *Salmonella* in the presence of the two drugs was measured at an excitation/emission wavelength of 622/670 nm, with an interval of 3 min for 30 min.

### 4.8. Scanning Electron Microscopy

*Salmonella* was cultured to the logarithmic phase (108 CFU/mL) in MHB medium. Cinnamaldehyde or ceftriaxone sodium was added to obtain different final concentrations, and the culture was incubated in a shaking incubator at 37 °C. An appropriate amount of the bacterial culture was added at 12 h and centrifuged at 4500 rpm for 10 min. The bacterial pellet was collected and washed three times with sterile PBS until the supernatant was transparent and colourless, after which the supernatant was discarded. The cells were fixed in 2.5% glutaraldehyde solution overnight at 4 °C, gently washed thrice with PBS, and dehydrated with 30%, 50%, 70%, 90%, and 100% ethanol solutions. Each sample was treated for 5 min, dried at room temperature (20–30 °C), and sprayed with gold. Bacterial morphology was observed under a scanning electron microscope [52].

### 4.9. Bacterial Viability Assay

The Live/Dead bacterial viability kit (catalogue no. L7007; Invitrogen) was used to evaluate cytotoxicity induced by the combination of cinnamaldehyde and ceftriaxone sodium. The drug-resistant *Salmonella* culture was washed three times and resuspended in 0.01 M PBS, and the OD600 nm value was adjusted to approximately 0.1. Next, different concentrations of cinnamaldehyde and sub-bacteriostatic ceftriaxone sodium (1024 µg/mL) were added to the solutions containing an equal number of bacteria. After 1 h of incubation at 37 °C, bacteria were collected, washed, and resuspended in PBS. In a microcentrifuge tube, 1.5 μL of 1.67 M SYTO9 (Thermo Fisher Scientific, Shanghai, China) and 1.5 μL of 10 mM PI (Thermo Fisher Scientific, China) were mixed thoroughly. Then, 3 μL of the mixed dye solution was added to each sample, followed by 15 min of incubation at room temperature (20–25 °C) in the dark. Five microlitres of the stained bacterial suspension were placed on a glass slide and secured with a coverslip, and the bacterial images were captured using a fluorescence microscope (Olympus Corporation, Tokyo, Japan) [23].

### 4.10. Antibiotic Accumulation Analysis

To generate a standard curve, an appropriate amount of ceftriaxone sodium standard was added with distilled water to prepare a stock solution (4 mg/mL), which was diluted to obtain a linear range (2–200 μg/mL) of ceftriaxone sodium. 

High-performance liquid chromatography (HPLC) was performed to detect and analyse the accumulation of antibiotics in drug-resistant *Salmonella* [30]. One millilitre of the drug-resistant *Salmonella* SJ2 was added to 100 mL fresh TSB medium and cultured overnight at 37 °C to reach OD600 nm = 0.6 [13]. Next, bacterial cells were collected and resuspended in fresh PBS, and then cinnamaldehyde and ceftriaxone were added at different concentrations and the samples were incubated at 37 °C for approximately 30 min in a shaking incubator. After incubation, the samples were centrifuged at 13,000× *g* for 2 min to precipitate the cells. To lyse the cells, each precipitate was dissolved in 400 μL sterile water, followed by three freeze-thaw cycles performed in liquid nitrogen and a 65 °C water bath. Next, the lysate was precipitated at 13,000× *g* for 2 min, and supernatant 1 was collected, resuspended in 200 μL methanol, and centrifuged under the same conditions, wherein supernatant 2 was collected. Supernatants 1 and 2 were mixed, the residue was removed via centrifugation at 13,000× *g* for 10 min, and the supernatant was collected. The final supernatant was analysed using HPLC (Agilent, Santa Clara, CA, USA). A Phenomenex Gemini C18 column (4.6 mm × 150 mm, 5 μm) was used to prepare the mobile phase. Briefly, Na_2_HPO_4_ (7.1 g) and KH_2_PO_4_ (6.8 g) were dissolved separately in 1L ultrapure water, and KH_2_PO_4_ was used to adjust the pH value of the Na_2_HPO_4_ solution between 7.6–7.8. The pH-adjusted solution was mixed with acetonitrile in a ratio of 88:12 to form the final mobile phase solution. The flow rate was 1.0 mL/min, injection volume was 10 μL, detection wavelength was 254 nm, and sampling time for each sample was 10 min.

### 4.11. Cephalosporin Hydrolysis Analysis

A single colony was cultured overnight in MH medium to allow it to reach the logarithmic phase. An aliquot of the bacterial culture (100 μL) was added to 30 mL MH medium. Different concentrations of the drugs were added to the medium, and cultures were divided as follows: bacterial liquid control group, sub-inhibitory concentration cinnamaldehyde (64 μg/mL) group, sub-inhibitory concentration ceftriaxone sodium (1024 μg/mL) group, and combined medication group. Bacterial cultures in each group were incubated for at least 4 h in a shaking incubator at 37 °C and sampled separately. The bacterial solutions were then diluted to achieve the same OD value and centrifuged at 6700 g for 10 min at 4 °C. The bacterial cells were collected, resuspended in 0.1 M PBS, and centrifuged for 10 min under the same conditions. Finally, the bacterial cells were resuspended in 0.01 M PBS and subjected to ultrasonic disruption in an ice water bath (650 W, power at 100%, 11 min 15 s, output 6 s, and interval 3 s). The sonicated cells were centrifuged for 1 h at 11,000 g and 4 °C, and the supernatant containing β-lactamase was obtained. Culture supernatant (50 μL) and cephalosporin (1 mg/mL, 50 μL; Yuanye Biological Technology Co., Ltd., Shanghai, China) were added to each group in a 96-well plate and incubated at 37 °C for 10 min. Once a change in colour was observed, the absorbance was measured at 490 nm and compared with the control group of bacteria to detect the hydrolysis rate of cephalosporin in each group using the following formula:

$$\mathrm{Hydrolysis}\;\mathrm{rate}=\left(\frac{OD_{490}\mathrm{of}\;\mathrm{each}\;\mathrm{group}}{OD_{490}\mathrm{of}\;\mathrm{control}\;\mathrm{group}}\right)\times 100\%$$

### 4.12. SDS-PAGE

The enzyme-containing supernatants from different groups and a 4× loading buffer were separately mixed at a ratio of 1:3 and denatured at 98 °C for 5 min. Then, 15 μL of each sample was used for electrophoresis. The samples included protein markers, pure β-lactamase (Macklin, Shanghai, China), bacterial samples from the control group, sub-inhibitory concentrations of cinnamaldehyde, the ceftriaxone sodium combination group, and the sub-inhibitory concentration cinnamaldehyde group alone. Electrophoresis was performed at 120 V for 90 min, and the concentrations of the stacking and resolving gels were 3% and 10%, respectively. After electrophoresis, the gel was stained with Coomassie Brilliant Blue R250 overnight, destained for 4–8 h, and photographed using a gel imager.

### 4.13. Inhibition of Enzyme Activity

A single colony was selected and cultured in MH medium overnight to the logarithmic phase. The method described above was used to extract the bacterial β-lactamase from the supernatant. The enzyme-containing supernatant was divided into a control group and a sub-inhibitory concentration of cinnamaldehyde group (cinnamaldehyde was added to the enzyme-containing supernatant to obtain a final drug concentration of 64 μg/mL), and a sub-inhibitory concentration of ceftriaxone sodium group (ceftriaxone sodium was added to the enzyme-containing supernatant to obtain a final drug concentration of 1024 μg/mL). After mixing the enzyme extract and drug, 50 μL of the mixture and 50 μL of cephalosporin were mixed and incubated at 37 °C for 30 min, and the OD490 was measured. A lower OD value indicated lower enzyme activity, reflecting the inhibitory effect of the combination medication on enzyme activity.

### 4.14. RT-PCR for Salmonella β-lactamase

*Salmonella* was grown overnight in LB broth and diluted 1:100 in 1 mL fresh LB supplemented with ceftriaxone sodium (1024 µg/mL) alone or in combination with cinnamaldehyde (32–128 μg/mL). After the bacterial cells were grown to mid-log phase (OD600 = 0.5) at 37 °C, total RNA was extracted using a bacterial total RNA extraction kit (Tiangmo Biotech, Beijing, China) and quantified using a Nanodrop spectrophotometer (Thermo Scientific, MA, USA) based on the ratio of absorbance at 260 nm and 280 nm. 

For cDNA synthesis, an equivalent amount of RNA was extracted from all samples. The extracted RNA was reverse-transcribed using 5× All-In-One MasterMix (abm, Vancouver, BC, Canada) [53]. The reverse transcription reaction conditions were as follows: 37 °C, 15 min, 85 °C for 5 s, and 4 °C incubation. PCR was performed in a 20 μL volume containing EvaGreen 2× qPCR MasterMix-No Dye (abm, Vancouver, BC, Canada), according to the manufacturer’s instructions. The primer sequences were used in this study showed in Table 2. The PCR program was set as follows: 95 °C for 3 min, 95 °C for 5 s, Tm for 30 s, a total of 39 cycles, 95 °C for 10 s, melting curve speed of 0.5 °C/5 s, 65–95 °C. The annealing temperature was set according to the actual annealing temperature of each primer. Once reverse-transcribed, the cDNA was stored at −20 °C. The expression of the target transcripts was calculated relative to that of *16S* rRNA (housekeeping gene) using the 2−ΔΔCt method [54].

### 4.15. Transcriptomic Analysis

Ceftriaxone sodium-resistant *Salmonella* SJ2 was grown in MHB medium until the exponential phase. The cells were then incubated with ceftriaxone sodium (1024 µg/mL) alone or in combination with cinnamaldehyde (64 µg/mL) for 4 h. Total RNA was degraded using TRIzol^®^ reagent according to the manufacturer’s instructions (Invitrogen), and genomic DNA was extracted using DNase I (Takara). RNA quality was determined using a 2100 Bioanalyzer (Agilent) and quantified using ND-2000 (NanoDrop Technologies). Only high-quality RNA samples (OD260/280) were used to construct the sequencing library. The subsequent steps were completed using the method described by Stokes et al. [47].

### 4.16. Animal Experiment

SPF KM mice (6–8 weeks old) were obtained from the Chengdu Dashuo Company. The mice were adapted to standardised environmental conditions (23 ± 2 °C, 55 ± 10%) for one week prior to infection. The mice were maintained in strict accordance with the regulations of the Administration of Affairs Concerning Experimental Animals approved by the State Council of the People’s Republic of China (14 November 1988). Animal experiments were performed in accordance with the relevant guidelines and regulations. The laboratory animal usage licence number SCXK-2020-030 was certified by the Sichuan Provincial Laboratory Animal Management Committee. All animals were maintained in a pathogen-free environment and fed ad libitum. The procedures for care and use of animals were approved by the Ethics Committee of Sichuan Agricultural University, and all applicable institutional and governmental regulations concerning the ethical use of animals were followed.

In general, acute peritonitis caused by bacterial infection is called bacterial peritonitis. The bacterial peritonitis model induced by drug-resistant *Salmonella* SJ2 was established with reference to the method of Lozano et al. [55]. SPF KM mice were intraperitoneally injected with 1.0 × 108 CFU of *Salmonella* for survival rates (*n* = 10 per group), and 0.5 × 108 CFU of *Salmonella* for the determination of colonization and inflammatory cytokines (*n* = 6 per group). Three hours post-infection, the mice were treated with a single dose of ceftriaxone sodium (200 mg/kg), cinnamaldehyde (50 mg/kg), or a combination of ceftriaxone sodium and cinnamaldehyde (50 and 200 mg/kg, respectively) via intramuscular injection and gavage. Separate negative control group (no *Salmonella*), positive control group (only infected with *Salmonella*). The survival rates of the treated mice were recorded for five days. At 1, 3 day post infection, 3 mice in each group were killed for determination of colonization and inflammatory cytokines. Total RNA was extracted following the manufacturer’s instructions using a specialized kit. The DNA sequences of the PCR primers were showed in Table 3. Relative gene expression was calculated by the ratio of the target gene to reference gene (*β-actin*) expression using the 2−ΔΔCt method [54].

### 4.17. Statistical Analysis

All experiments, except for the animal assays, were repeated at least three times, and the average value of all experiments was used. All data are presented as the mean ± standard deviation (SD), and the significance of differences was analysed using an unpaired two-tailed Student’s t-test or deviation analysis and analysis of variance (ANOVA) using GraphPad Prism 7 software. Differences were considered significant and extremely significant at *p* < 0.05 and *p* < 0.01, respectively.

## Figures and Tables

**Table 1:** Strain information and the MICs.

Strain Name	Identification	Strain Source	MIC of Ceftriaxone Sodium (μg/mL)	MIC of Cinnamalde-Hyde (μg/mL)	Antibiotic Resistance ^a^
ATCC14028	*Salmonella typhimurium*	Standard strain	0.5	128	\
ATCC13036	*Salmonella pullorum*	Standard strain	0.5	128	\
SJ2	*Salmonella enteritidis*	Duck	4096	256	AMP CRO CFZ CTX DOX KAN
3–30	*Salmonella enteritidis*	Chicken	4096	256	AMX CRO CTX
3–65	*Salmonella enteritidis*	Chicken	2048	256	CRO CFZ CTX
3–93	*Salmonella enteritidis*	Chicken	4096	512	AMX CRO CFZ
11–49	*Salmonella enteritidis*	Chicken	4096	256	AMP CRO CTX
EBCC	*Escherichia coli*	Chicken	1024	256	CRO GEN STR
Klebsiella pneumoniae	*Klebsiella pneumoniae*	Human	2048	512	AMP AMX CRO CFZ KAN
*Acinetobacter baumannii*	*Acinetobacter baumannii*	Chicken	8	256	AMP AMX CRO CTX DOX GEN KAN

^a^: The antibiotic resistance of 8 clinical isolates was determined by the Disc diffusion method. Inhibitory zone diameter of various antibiotics: Ampicillin (AMP), ≤16 mm; Amoxicillin (AMX), ≤13 mm; Ceftriaxone (CRO), ≤22 mm; Cefotaxime (CFZ), ≤ 20 mm; Amoxicillin (CTX), ≤19 mm; Doxycycline (DOX), ≤12 mm; Gentamicin (GEN), ≤12 mm; Streptomycin (STR), ≤12 mm; Kanamycin (KAN), ≤13 mm.

**Table 2:** Primer sequences with their corresponding PCR product length for β-lactamase.

Primer	Sequence (5′→3′)	Tm (°C)	Product
*16s-f*	GCTGCCCTTTGTATTGTC	56	1506 bp
*16s-r*	AGATGTTGGGTTAAGTCCC		
*TEM-f*	TCGCCGCATACACTATTCTCAGAATGA	60	800 bp
*TEM-r*	ACGCTCACCGGCTCCAGATTTAT		
*SHV-f*	GCCTTTATCGGCCTTCACTCAAG	60	898 bp
*SHV-r*	TTAGCGTTGCCAGTGCTCGATCA		
*CTX-M-f*	ACGCTTTCCAATGTGCAGTA	60	436 bp
*CTX-M-r*	ACGCTTTCCAATGTGCAGTA		
*CMY-F*	GACAGCCTCTTTCTCCACA	58	1201 bp
*CMY-R*	TGGAACGAAGGCTACGTA		
*OXA-F*	ATGAAAAACACAATACATATC	58	896 bp
*OXA-R*	CGTATAGGTGTTTCCGTTCT		

**Table 3:** Primer sequences with their corresponding PCR product length for aniamls experiment.

Gene	Primer (5′-3′)	Tm (°C)	Product
*β-actin-F*	CTACAGCTTCACCACCACAG	57	118 bp
*β-actin-R*	ACCGCTCGTTGCCAATAGTG		
*IL-6-F*	CTGCAAGAGACTTCCATCCAG	57	132b p
*IL-6-R*	AGTGGTATAGACAGGTCTGTTGG		
*IL-1β-F*	TGCCACCTTTTGACAGTGATG	56	117 bp
*IL-1β-R*	TGATACTGCCTGCCTGAAGC		
*IL-10-F*	GAGTGAAGACCAGCAAAGGC	55	168 bp
*IL-10-R*	TTGTCCAGCTGGTCCTT		
*TNF-α-F*	GCCACCACGCTCTTCTGTCTAC	58	283 bp
*TNF-α-R*	GGGTCTGGGCCATAGAACTGAT		
